# High traffic roads and adverse birth outcomes: comparing births upwind and downwind of the same road

**DOI:** 10.1093/aje/kwae120

**Published:** 2024-06-14

**Authors:** Andrew Larkin, Mary D Willis, Lena Harris, Beate Ritz, Elaine L Hill, Perry Hystad

**Affiliations:** School of Nutrition and Public Health, College of Health, Oregon State University, Corvallis, OR 97331, United States; Department of Epidemiology, School of Public Health, Boston University, Boston, MA 02118, United States; Department of Economics, School of Arts and Sciences, University of Rochester, Rochester, NY 14627, United States; Department of Epidemiology, Fielding School of Public Health, University of California, Los Angeles, CA 90095, United States; Department of Economics, School of Arts and Sciences, University of Rochester, Rochester, NY 14627, United States; School of Nutrition and Public Health, College of Health, Oregon State University, Corvallis, OR 97331, United States

**Keywords:** air pollution, birth outcomes, traffic, wind, instrumental variable

## Abstract

Traffic-related air pollution is a major concern for perinatal health. Determining causal associations, however, is difficult because high-traffic areas tend to correspond with lower socioeconomic neighborhoods and other environmental exposures. To overcome confounding, we compared pregnant individuals living downwind and upwind of the same high-traffic road. We leveraged vital statistics data for Texas from 2007 to 2016 (*n* = 3 570 272 births) and computed hourly wind estimates for residential addresses within 500 m of high-traffic roads (ie, annual average daily traffic >25 000 vehicles) (10.9% of births). We matched pregnant individuals predominantly upwind with pregnant neighbors downwind of the same road segment (*n* = 37 631 pairs). Living downwind was associated with a decrease of 11.6 g (95% CI, -18.01 to -5.21) in term birth weight. No associations were observed with low term birth weight, preterm birth, or very preterm birth. In distance-stratified models, living downwind within 50 m was associated with a decrease of 36.3 g (95% CI, -67.74 to -4.93) in term birth weight and living 51-100 m downwind was associated with an odds ratio of 3.68 (95% CI, 1.71-7.90) for very preterm birth. These results suggest traffic air pollution is associated with adverse birth outcomes, with steep distance decay gradients around major roads.

This article is part of a Special Collection on Environmental Epidemiology.

## Introduction

Traffic-related air pollution (TRAP) emissions have been associated with numerous adverse birth and early life health outcomes, including low term birth weight (TBW),^1^^,^^2^ increased odds of preterm birth (PTB),^1^^,^^3^ cardiac^4^^,^^5^ and neural tube birth defects,^4^ and increased rates of childhood asthma incidence^6^ and hospital admissions.^7^ With a projected 22% increase in vehicle-miles traveled (VMT) over the next 30 years,^8^ traffic is likely to be a persistent concern for perinatal health for the foreseeable future.

Although there is evidence that TRAP is harmful to perinatal health, many challenges remain in quantifying its causal relationship with adverse birth outcomes. Traffic-related exposures, in general, represent a diverse combination of air pollutants^9^ (eg, brake dust,^2^ nitrogen oxides,^1^^,^^3^^,^^5^ particulates) and other environmental exposures (eg, noise,^2^^,^^10^ light pollution,^11^ neighborhood context^10^). These exposures are negatively correlated with distance to roadways,^12^ positively correlated with VMT,^13^ and highly correlated spatially and temporally with each other. Road density, VMT, and traffic emissions are also correlated with socioeconomic status,^12^ with persistently marginalized communities (eg, lower income, race/ethnicity) experiencing disproportionately greater exposures.^14^ These co-occurring conditions create a challenging source of confounding that is often an issue in epidemiologic studies of traffic air pollution. Given the rapid conversion of the vehicle fleet to electric vehicles over the next decade,^15^ determining the contribution of TRAP to perinatal health outcomes is central to policy and planning.

Wind can be used as an instrumental variable to disentangle TRAP effects on adverse birth outcomes from other traffic co-exposures and sociodemographic confounders. Neighbors living on the same street but in opposite wind direction are likely to experience similar non–air pollution exposures (because wind direction related to a road is unlikely to be associated with residential selection) but significantly differing air pollution exposures. Wind, therefore, satisfies the 2 key requirements for an instrumental variable: it determines a component of TRAP exposure and it is not directly related to health outcomes. However, only a few studies have integrated wind as an instrument into epidemiologic analyses.^16^^-^^19^ For example, in 1 study, researchers examined living downwind of highways in Los Angeles, CA, and mortality rates at the census block level, and observed that a doubling of the percentage of time spent downwind of a highway increased the mortality rate among individuals aged 75 years or older by 3.9%-6.4%.^19^

To disentangle TRAP from socioeconomic status and other environmental exposures, we matched maternal residence neighbors living upwind and downwind (within 500 m) of the same high-traffic road (> 25 000 vehicles in annual average daily traffic [AADT]) from 2007 to 2016 (*n* = 388 316 births). We developed and implemented an exposure assessment approach to estimate the percentage of time each pregnant person in the study was downwind of roadways and matched maternal residences predominantly upwind and downwind of the same high-traffic road, taking building and tree shielding into account. We then determined differences in TBW, low TBW, PTB, and very PTB.

## Methods

### Study population

We examined all births in Texas from 2007 to 2016, using vital statistics records (*n* = 3 570 272 births). Individual data from the vital statistics records were used to assess maternal sociodemographic characteristics, risk and protective factors for birth outcomes, and birth outcome measures (eg, birth weight, estimated gestational length). The participants’ residential addresses at time of delivery were used to assess TRAP exposures and additional environmental and contextual exposure measures. This study was approved by the institutional review board at Oregon State University (approval no. 6843) and the Texas Department of State Health Services (approval no. 15-029).

### Traffic air pollution and wind analyses

#### High-traffic roads

We identified high-traffic roads using annual traffic volumes from historical Texas Roadway Inventory databases.^20^ Roads with AADT ≥ 25 000 (ie, highways, expressways, and arterial roads with substantial traffic) were classified as high-traffic roads and included in the study. We then restricted our study population to birth residences within 500 m of high-traffic roads (based on year of birth) (Figure S1). We did not observe any notable differences in high-traffic road density near residences when classifying roads based on AADT levels during conception compared with birth. High-traffic roads were divided into 10-m road segments for subsequent wind analyses.

#### Wind exposure measures

**Figure 1:**
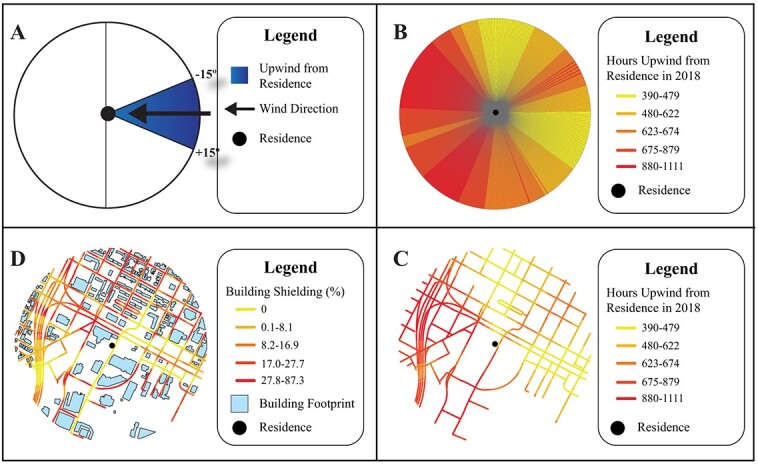
Estimating wind exposure and building shielding at a maternal residence. (A) Wind direction is converted into a 31° interval. All objects within the interval are classified as upwind of the residence. (B) Hourly intervals are summed over the length of pregnancy, creating 360 radial segments. To protect cohort privacy, intervals are summed over 2018 for US Environmental Protection Agency air monitor EP_10-march 1, 2004. (C) Radial segments were spatially joined to road networks to estimate hours for each road. Segment was upwind from residence. (D) Buildings between roads and the residence contributed to building shielding. The radii for all circles are 500 m. Variables in B-D are colored by quantile.

We downloaded hourly u and v wind vectors from 2007 to 2016 from the European Centre for Medium-Range Weather Forecasts (ECMWF; fifth major global reanalysis produced by ECMWF [ERA5] reanalysis version 5).^21^ For each maternal residence, we calculated the radial distribution of hourly wind directions during pregnancy at 1 radial-degree resolution from 0:00 on the estimated date of conception until 23:00 on the birth date. We then applied a ±15° radial interval and estimated the number of hours during pregnancy the maternal residence was downwind of all high-traffic roads within each 1° radial segment (eqn (1), Figure 1A). In eqn (1),


(1) \begin{equation*} {d}_{ij}=\sum_{k_i=i}^{m_i}\left\{\begin{array}{c}1\ if\ wind\ direction\ is\ within\ 15\ radial\ degrees\\{}0\ otherwise\end{array}\right. \end{equation*}

*d_ij_* is the number of hours the maternal residence *i* is downwind of high-traffic roads located within radial segment *j* during pregnancy; *k_i_* is the *k*th hour of pregnancy for maternal residence *i*; and *m_i_* is the number of hours during pregnancy (conception until birth) for the pregnant woman at residence *i*. This process was performed for 360 radial segments, thus providing complete radial coverage of each residence (Figure 1B).

#### Hours downwind of high-traffic roads

For each maternal residence, the wind direction radial segments were spatially joined with the high-traffic road data set described above to estimate the number of hours from estimated date of conception until birth that the residence was downwind of each 10-m high-traffic road segment within 500 m (Figure 1C). We then calculated the mean hours and percentage of each pregnancy when the residence was downwind from high-traffic roads (eqn 2), identified the road segment that was upwind the greatest number of hours from the residence (maximum [max] upwind road; eqn (4)), and then calculated the hours and percentage of pregnancy that the residence was downwind from the max upwind road (eqn 3). Equations (2)-(4) are as follows:


(2) \begin{equation*} mean\ hours\ downwin{d}_i=\frac{1}{n_i}\sum_{l_i=1}^{n_i}{d}_{l_i} \end{equation*}

where mean hours downwind*_i_* means number of hours maternal residence *i* is downwind of 10-m road segments within 500 m during pregnancy (conception until birth); *n_i_* is the number of high-traffic road segments within 500 m of residence *i*; *l_i_* is the *l*th 10-m high-traffic road segment within 500 m of residence *i*; and *d_li_* is the number of hours during pregnancy that residence *i* is downwind of *l_i_*.


(3) \begin{align*}downwind\ from\ max\ {road}_i=\mathit{\max}\Big(\left\{{d}_{li}\right\}\Big)\end{align*}

where {*d_li_*} is the set of *dli* values described in eqn (2) for all road segments *l* within 500 m of residence *i*. And


(4) \begin{equation*} \mathit{\max}\ roa{d}_i=f \end{equation*}

where max road*_i_* is the road segment with the greatest number of hours upwind from residence *i*; *f(x)* is the function that returns the identification of road segment *li* given input value *dli*, as described in eqn (2). If there are multiple road segments associated with input *x*, *f(x)* returns the identification of the road segment closest to the maternal residence.

#### Building and tree shielding

In addition to wind direction, shielding by buildings or trees may affect air pollution dispersion. Annual Texas building footprints (2007-2016) were developed by combining Microsoft Bing building footprints,^22^ land parcel records from the Texas Natural Resource Information System,^23^ and building records from CoreLogic^24^ (Appendix S1). Using the road segments described above, we calculated the percent area between road segment and maternal residence occupied by a building footprint (Figure 1D).

#### Matching downwind (exposed) and upwind (control) maternal residences

**Figure 2:**
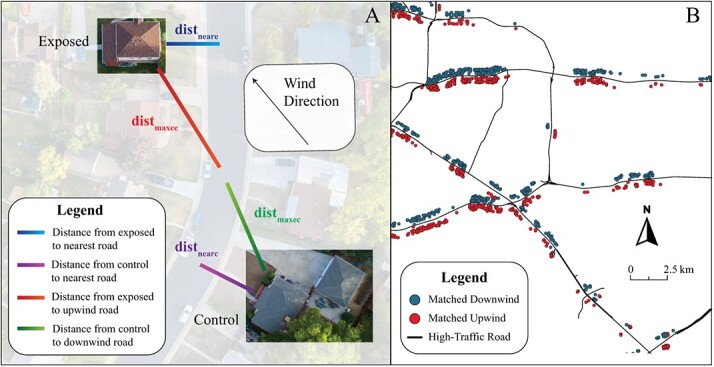
Matching downwind and upwind residences in Texas from 2007 to 2016. (A) Matching maximizes the similarity in distance to road between exposed (downwind) and control (upwind) residences. (B) Matched residences are mostly neighbors on opposite sides of a high-traffic road.

Residents in the top and bottom quartiles of hours downwind from max road (Eq 3) were categorized as exposed (ie, top quartile downwind; *n* = 97 024) and control (ie, bottom quartile downwind, referred to hereafter as the upwind group; *n* = 97 026) groups. Exposed residences were then matched with up to 4 control residences (median = 2), giving priority to matches with the lowest match score, calculated using eqns (5)-(8) (Figure 2A). The match score estimates the difference in road and neighborhood-level exposures between exposed and control addresses, except for wind direction. Approximately 98% of matches were neighbors on opposite sides of high-traffic roads (Figure 2B). Equations (5)-(7) are as follows:


(5) \begin{equation*} locality\ score= abs\left( dis{t}_{maxee}- dis{t}_{maxec}\right) \end{equation*}

where locality score is the difference in distance to the matched road between exposed and control residences; *dist_maxee_* is the distance from exposed residence to the max road segment (eqn 3) for exposed residence *e*; and *dist_maxec_* is the distance from control residence to the max road segment (eqn 3) for exposed residence *e*.


(6) \begin{equation*} near\ road\ score= abs\left( dis{t}_{neare}- dis{t}_{nearc}\right) \end{equation*}

where the near road score equals the similarity between exposed and control residences in distance to nearest road; *dist_neare_* is the distance from nearest road to the exposed residence; and *dist_nearc_* is the distance from nearest road to the control residence.


(7) \begin{align*} match\ score&= locality\ score+ near\ road\ score\nonumber\\&+10\ x\ abs\left( birth\ yea{r}_e- birth\ yea{r}_c\right) \end{align*}

where birth year*_e_* is the birth year at the exposed residence; birth year*_c_* is the birth year at the control residence; and birth year*_c_* must be ± 4 years of birth year*_e_*.

Matches were restricted to within 4 years’ difference in birth year to limit unmeasured confounding arising from neighborhood development over time. We selected 4 years because further restricting match criteria to within 3 years reduced the number of matches by 12%, whereas relaxing birth year match criteria to within 5 years increased the number of matches by only 2%.

A final distance-based inclusion criterion (eqn 8) was applied to matches before proceeding to epidemiologic analyses:


(8) \begin{equation*} inlusion\ criteria=\left\{\begin{array}{@{}c}1\ if\ locality\ score<100\ and\ near\ road\ score<100\\{}0\ otherwise\end{array}\right. \end{equation*}

Matches were excluded if the exposed and control residences were too dissimilar with respect to either the distance to nearest road or distance to the exposed max downwind road. Computer scripts for implementing the wind exposure assessment methods are available at www.github.com/larkinandy/Matching_HEI_4970.

### Epidemiological analysis

We tested for differences between matched upwind and downwind residences in TBW (delivery at 37-42 weeks’ gestation), low TBW (TBW < 2500 g), PTB (22-37 weeks gestational age), and very PTB (22-32 weeks gestational age) using linear and logistic regression. For TBW and low TBW outcomes, records were restricted to 37-42 weeks estimated gestational age before creating matched pairs. For each outcome, we created 2 regression models using 2 covariate sets. The base set of covariates included infant sex (female, male), maternal age (linear), birth month (categorical), year (categorical), distance to nearest road (linear), and county (categorical). The base model for TBW also included estimated weeks of gestation (categorical). The full set of covariates included the base model plus maternal race and ethnicity (see Table 1 for all categories); whether the mother was born outside of the United States (yes, no); source of payment used for delivery (ie, Medicaid, private insurance, self-pay, or other); maternal use of the Women, Infants, and Children program during pregnancy (yes, no); maternal smoking status during pregnancy (yes, no); maternal weight gain (linear); month that pregnancy prenatal care began (none, months 1-9); and neighborhood income (tertiles by year). We ran models for all matched pairs and then tested for interactions between wind direction and air pollution decay gradients by stratifying regression models by distance to the nearest road.

**Table 1:** Descriptive statistics for matched pairs of pregnant individuals (*n* = 41 912 pairs) living predominantly downwind and upwind of the same major road.

	**Predominantly downwind**	**Predominantly upwind**
No. of pairs	40 693	40 693
Downwind from max road^a^^,^^b^ (%)	24.6	7.0
Mean downwind (%)	11.3	4.6
Max tree shielding (%)	4.1	4.1
Max building shielding (%)	10.8	10.3
Estimated gestational age (weeks)	38.4	38.4
Term birth weight (g)	3243	3260
No. of pairs with low term birth weight	3009	2881
No. of pairs with preterm birth	3801	3710
No. of pairs with very preterm birth (n)	559	555
Insurance		
Medicaid insurance (%)	50.4	48.2
Private insurance (%)	28.9	32.0
Self-pay insurance (%)	12.2	11.7
Other insurance (%)	8.3	7.9
Race/ethnicity (%)		
White	64.0	66.3
Black	15.5	13.9
Native American	0.23	0.2
Asian	4.8	5.3
Pacific Islander	0.1	0.2
Other	14.4	13.3
Non-Hispanic/Latina	42.9	44.2
Hispanic	57.2	55.8
Education level (%)		
Less than 8th grade	6.9	6.5
Up to high school diploma	51.6	48.6
Up to bachelor’s degree	35.4	37.7
More than a bachelor's degree	6	7.1
Reported smoking	5.2	5.1
Neighborhood income tertile		
Low	33.3	33.3
Middle	33.3	33.3
High	33.3	33.3
Mother was born in the United States	66.1	67.1
Mother was born outside of the United States	33.9	32.9

^a^ Max road refers to the road segment with the greatest number of hours upwind from a given residence.

^b^ Max, maximum.

### Sensitivity analyses

We conducted several sensitivity analyses to examine our wind exposure measures, matching methods, and potential effect modification by environmental and individual measures. First, we conducted stratified analyses by race, ethnicity, income, education, and whether the mother was born in the United States. Next, we substituted the categorical exposed/control variable with the continuous variable of percent downwind from max road (eqn 3). Third, we included our building and shielding metrics. Fourth, we implemented more stringent inclusion matching criteria (15, 25, and 50 m) compared with the 100 m used in our main analyses. Finally, we conducted analyses using 3-year rolling averages to evaluate temporal trends in our epidemiologic models.

## Results

### Descriptive statistics

Characteristics of matched exposed and control birth addresses are shown in Table 1. Statistics for matched pairs restricted to 37-42 weeks estimated gestational age are available in Table S1. Exposed residences were, on average, downwind from the max downwind road (eqn 3) 3.5 times longer than their upwind counterparts (24.6% vs 7.0%) during pregnancy. Exposed residences were downwind from the average road segment 2.5 times longer than upwind matches (11.3% vs 4.6%). Building and tree shielding were similar between downwind and upwind residences. The percentages of pregnant people who were Black, Hispanic and/or had a lower level of education were slightly greater among paired downwind residences compared with paired upwind residences.

### Linear and logistic regression models

Results of the base and fully adjusted linear and logistic regression models are shown in Table 2 and Figure S2. Living downwind was associated with a 13.8 g (95% CI, -20.03 to -7.63) and 11.6 g (95% CI, -18.01 to -5.21) decrease in TBW in the base and fully adjusted models, respectively. There were no significant associations with living downwind and low TBW, PTB, or very PTB.

**Table 2:** Relationships between birth outcomes and living predominantly downwind compared with upwind of major roads.

**Outcome**	**No. of pairs**	**No. of events** ^a^	**Base model** ^b^	**Full model** ^c^
Term birth weight^d^	37 631	NA^e^	-13.80 (-20.03 to -7.63)	-11.61 (-18.01 to -5.21)
Low term birth weight^f^	37 631	2049	1.00 (0.91-1.10)	0.98 (0.88-1.09)
Preterm birth^f^	40 693	7511	1.01 (0.96-1.06)	1.01 (0.96-1.07)
Very preterm birth^f^	40 693	1114	0.96 (0.84-1.09)	1.03 (0.87-1.23)

^a^ Events are total number of events, not pairs with an event.

^b^ Base model: wind exposure (upwind is reference), birth sex, maternal age, birth month and year, distance to nearest road, and a fixed effect for county. The base model for term birth also included estimated gestational age.

^c^ Full model: base model + maternal race and ethnicity; whether mother was born outside of the United States; source of payment used for delivery; maternal Women, Infants, and Children program use during pregnancy; maternal smoking status during pregnancy; maternal weight gain; month of pregnancy prenatal care began; and neighborhood income.

^d^ Term birth weight: β reported in grams (95% CI).

^e^ NA, not applicable;

^f^ Low term birth weight, preterm birth, and very preterm birth reported as odds ratio (95% CI).

#### Stratifying by distance to nearest road

Associations between birth outcomes and wind direction when stratifying by distance to nearest road are summarized in Table 3 (see Table S2 for additional details). Residences downwind and within 50 m of high-traffic roads had significantly lower TBW by -36.3 g (95% CI, -67.74 to -4.93) and statistically nonsignificant increased odds of low TBW (odds ratio [OR] = 1.95; 95% CI, 0.58-6.51) and PTB (OR = 1.25; 95% CI, 0.92-1.69) compared with upwind residences similarly within 50 m of the road. Models for very PTB did not converge for the 0-50 m group, but for 51 to 100 m, the OR for living downwind compared with upwind was 3.68 (95% CI, 1.71-7.90).

**Table 3:** Relationships between birth outcomes and living predominantly downwind compared with upwind of major roads, stratified by distance to the nearest major road.

**Distance (m)**	**Term birth weight (g)**	**Low term birth weight (OR)** ^a^	**Preterm birth (OR)**	**Very preterm birth (OR)**
0-50	-36.33 (-67.74 to -4.93)	1.95 (0.58-6.51)	1.25 (0.92-1.69)	Did not converge
51-100	-17.59 (-36.32 to 1.15)	1.44 (0.91-2.29)	1.18 (0.99-1.40)	3.68 (1.71-7.90)
101-300	-13.97 (-22.15 to -5.79)	1.03 (0.89-1.18)	1.05 (0.98-1.13)	1.14 (0.91-1.43)
301-400	-15.55 (-24.97 to -4.13)	0.93 (0.77-1.12)	1.04 (0.95-1.14)	1.11 (0.81-1.52)
401-500	4.02 (-10.66 to 18.71)	0.96 (0.72-1.26)	0.99 (0.87-1.14)	1.01 (0.61-1.67)

^a^ OR, odds ratio.

#### Sensitivity analyses

Associations between birth outcomes and wind direction stratified by select sociodemographic characteristics are summarized in Table 4. Additional details are in Tables S3 and S4. Living downwind was associated with 1.21 (95% CI, 0.69-2.12), and 1.21 (95% CI, 0.94-1.56), and 2.92 (95% CI, 1.00-8.53) increased odds of low TBW, PTB, and very PTB, respectively, for Black non-Hispanic births. However, Black non-Hispanic births associated with residences downwind also were associated with a statistically nonsignificant 20.7 g (95% CI, -10.80 to 52.21) increased TBW. Lower level of education (high school or less) was associated with increased odds of PTB (OR = 2.69; 95% CI, 0.52-13.88), low TBW (OR = 1.85; 95% CI, 1.18-2.88), and decreased TBW (-23 g; 95% CI, -43.51 to -2.54). Residents with a high income and living downwind had decreased TBW (-13.59 g; 95% CI, -27.55 to 0.36) and decreased odds of very PTB (OR = 0.53; 95% CI, 0.29-0.95) and low TBW (OR = 0.77; 95% CI, 0.58-1.03).

**Table 4:** Associations between birth outcomes and living predominantly downwind compared with upwind of major roads, stratified by select sociodemographic characteristics.

	**Term birth weight (g)**	**Low term birth weight (OR)** ^a^	**Preterm birth (OR)**	**Very preterm birth (OR)**
Race/ethnicity				
Black non-Hispanic	20.70 (-10.80 to 52.21)	1.21 (0.69-2.12)	1.21 (0.94-1.56)	2.92 (1.00-8.53)
White non-Hispanic	-5.93 (-25.77 to 13.87)	0.87 (0.48-1.58)	1.05 (0.77-1.18)	Did not converge
Hispanic or Latina	-8.02 (-20.41 to 4.37)	0.99 (0.80-1.24)	1.02 (0.88-1.10)	1.18 (0.82-1.73)
Education level				
High school or less	-23.02 (-43.51 to -2.54)	1.85 (1.18-2.88)	1.11 (0.92-1.34)	2.69 (0.52-13.88)
More than high school	-8.70 (-17.32 to -0.08)	0.97 (0.83-1.12)	0.99 (0.92-1.07)	1.02 (0.79-1.32)
US born	-12.87 (-22.23 to -3.50)	0.98 (0.84-1.14)	0.99 (0.91-1.07)	1.06 (0.82-1.36)
Foreign born	-8.63 (-25.89 to 8.63)	0.92 (0.60-1.42)	1.16 (0.96-1.39)	1.23 (0.39-3.87)
Low income	1.73 (-12.42 to 15.88)	1.04 (0.79-1.35)	1.14 (1.01-1.28)	1.15 (0.75-1.74)
High income	-13.59 (-27.55 to 0.36)	0.77 (0.58-1.03)	0.97 (0.85-1.11)	0.53 (0.29-0.95)

^a^ OR, odds ratio.

When examining different match criteria, we observed larger associations with more restrictive matches (Table S4). When replacing the exposure group with a continuous wind variable, for every 10% increase in the number of hours spent downwind, TBW decreased by -4.96 g (95% CI, -8.43 to -1.50) (Table S5). Inclusion of the shielding metrics did not change our wind estimates (Table S6). We did not observe any clear temporal patterns when evaluating rolling 3-year cohort subsets (Table S7).

## Discussion

This study contributes to the body of studies that use causal inference methods for estimating associations between TRAP and population health. Specifically, we leveraged wind as an instrumental variable for TRAP to disentangle the associations between TRAP and adverse birth outcomes from socioeconomic status–related confounding factors and other traffic-related environmental exposures (eg, noise) associated with residential locations near major roadways. We compared neighbors living on opposite sides of high-traffic roads. Living downwind was associated with an 11.6 g (95% CI, -18.01 to -5.21) decrease in TBW, with larger associations for individuals living downwind and within 100-300 m of the major road.

Our results of a positive association between TRAP exposure and adverse birth outcomes correspond to findings in the existing literature; however, the interpretation of our findings is different. Nearly the entire body of literature examining TRAP relies on comparing residential-based exposures over space and time, with most studies using road proximity or model-based predictors of TRAP (eg, from land-use regression models). Functionally, these studies often compare TRAP measures at residential addresses across entire cities, assessing exposure variation driven by local, neighborhood, and regional factors. Here, we restricted our study population to birth addresses within 500 m of high-traffic roads and used wind as an instrumental variable to differentiate TRAP exposure levels. Our finding of an 11.6 g (95% CI, -18.01 to -5.21) decrease in TBW for pregnant individuals living downwind compared with upwind of a matched road represented the impact of wind dispersion of air pollution alone within this heterogenous group. We also found a strong wind-distance interaction, with larger associations for individuals living downwind and within 100 m of the major road. A recent systematic review of 26 studies on traffic metrics (eg, road proximity or traffic density) and adverse birth outcomes^25^ found that the risk of term LBW was associated with traffic density in 500 m, with an estimated increased risk of 1.06 (95% CI, 1.002-1.121) in higher-quality studies. Studies have also reported that individuals living close to roads (< 50 and < 100 m) have larger risks (eg, LBW OR of 1.13; 95% CI, 1.04-1.21) for traffic density increases of 100 000 vehicles within 50 m,^26^ which corresponds to the wind-distance decay findings of our present study. Alternatively, a recent systematic review of TRAP concentrations found that a 5 μg m^-3^ increase in fine particulate matter was associated with a mean difference in TBW of -17.3 (95% CI, -33.2 to -1.5) g per 5 μg m^-3^, but no association was observed for NO_2._^27^ Our results add new information to this heterogeneous literature by demonstrating robust associations between TRAP exposure and TBW, especially very near large roadways.

Confounding of TRAP exposure by socioeconomic status and other environmental exposures related to traffic is a major concern in traffic-related epidemiologic studies. We used wind as an instrumental variable to compare individuals living upwind with those living downwind of the same road. A perfect instrument would be predictive of exposure (here, TRAP) but unrelated to individual and neighborhood characteristics. We observed small differences in our exposed (downwind) and control (upwind) groups for race, ethnicity, and education. However, these were much smaller (eg, generally < 2%-3%) compared with spatial differences in sociodemographic characteristics observed when comparing individuals near and far from roadways. For example, our previous research in Texas examining adverse birth outcomes for individuals < 300 m from highways or expressways, compared with individuals 500-1000 m from these types of roads, found differences of approximately 8% for less than high school education and White non-Hispanic pregnant individuals by near and far exposure status.^28^ We observed small attenuation in our base model to the fully adjusted model (eg, -13.80 to -11.61 g), but this is, again, much smaller than has been observed with traditional road-proximity studies. For example, in the unadjusted model comparing individuals < 300 m from highways or expressways with individuals 500-1000 m away, we observed a -29.5 g (95% CI, -33.1 to 26.0) decrease in TBW, which was attenuated to -7.5 g (95% CI, -10.9 to -4.1) with the inclusion of individual and neighborhood covariates.^28^ Our wind-based analysis, therefore, is less likely to suffer from residual or unmeasured confounding compared with traditional studies using road-proximity metrics.

Living downwind may increase exposure to TRAP in general, but susceptibility and competing risks may differ across race, ethnicity, income, education, and other sociodemographic characteristics. We conducted stratified analyses to examine differences by individual and neighborhood factors. These analyses suggested that Black non-Hispanic individuals may be particularly susceptible to increased risk for PTB and very PTB from this exposure (OR = 1.21 [95% CI, 0.94-1.56]; and OR = 2.92 [95% CI, 1.00-8.53], respectively). Competing risks may explain the increased TBW we observed among Black non-Hispanic residents living downwind: if susceptible Black non-Hispanic babies have an increased risk of being born early and thus being excluded from the TBW analysis, then there is a lower percentage of susceptible Black non-Hispanic babies who make it to full gestational age (and thus be included in the TBW analysis). For individuals with a high school diploma or lower level of education, we saw larger associations (eg, -23.0 g; 95% CI, -43.51 to -2.54) compared with individuals with more than a high school education (-8.70 g; (95% CI, -17.32 to -0.08). Surprisingly, individuals living in high-income neighborhoods had larger associations for TBW, but not for PTB or very PTB. However, the number of low TBWs in the high-income group was small (*n* = 368).

In this study, our wind exposure estimates assumed that maternal residences at birth were the primary exposure sites during pregnancy. This assumption does not hold for pregnant people who spent time away from home (eg, work) or lived at multiple residences.^29^ In this cohort, the number of participants who moved and/or worked during pregnancy is unknown. Violations of this assumption would result in overestimating exposure at an exposed ( ie, downwind) residence or underestimating exposure at a control (ie, upwind) residence. In either case, exposure misclassification arising from the violation of this assumption would result in underestimating the difference in exposure between an upwind/downwind matched pair and bias results toward the null. It is also possible that exposure estimates for the entire pregnancy were acting as secondary indicators for late pregnancy exposure (with a large percentage of moves during pregnancy) or evening/night exposure (with a large percentage of daytime workers).

This study has several limitations that should be acknowledged when interpreting our findings. First, wind exposures were derived from meteorological data that represent regional wind patterns, rather than localized wind direction. High-resolution models that incorporate local effects such as wind tunnels and turbulence may improve exposure estimates, especially within 50 m of high-traffic roads. Shielding covariates did not change associations between birth outcomes and living downwind. However, our shielding metrics were calculated independent of wind metrics and did not consider detailed street-level characteristics that may determine air dispersion.

The number of matches in our study is lower than expected within 50 m of high-traffic roads. This is a limitation of the study design because there was a small percentage of residences within 50 m of high-traffic roads that met the study criteria for a control classification. The closer a house is located next to a high-traffic road, the greater the range of wind directions that contribute to a downwind exposure (Figure S3). The matching algorithm may need to be modified for cohorts with small percentages of participants living near and on both sides of high-traffic roads, such as rural communities. The algorithm also relies on a consistent, long-term, prevalent wind direction.

Wind exposure estimates at maternal residences were derived using ERA5, which is a global product. The North American Regional Reanalysis is an alternative meteorological product optimized for North America and with similar spatial resolution (32 km). We chose to use the global ERA5 data set to maximize generalizability. However, ERA5 may have greater error and exposure misclassification at North American locations compared with the North American Regional Reanalysis. Our exposure methods use a ± 15° window for classifying road segments as upwind from residences (Figure 1). Small discrepancies between meteorological data sets should have minimal impacts on the estimated number of hours downwind from roads.

In this study, our matching score was optimized for creating the best possible geographic match between 2 participants with differing TRAP exposure (ie, 2 neighbors living upwind and downwind of a high-traffic road). Although we conceptualized wind as an instrumental variable, our matching algorithm did not explicitly consider or remove unmeasured confounding associated with demographics and economics, such as race and education. Restricting matches to the top and bottom quartiles of wind exposure greatly reduced sample size and statistical power, most notably in stratified sensitivity analyses. Transforming exposure measures from continuous to categorical also reduced exposure detail. Future studies could explore alternative matching designs that leverage continuous wind exposure metrics and integrate economic and demographic variables into propensity scores.^29^^-^^31^

Despite these limitations, this study provides strong evidence that living downwind of high-traffic roads has increased risk of adverse birth outcomes compared with living the same distance upwind; thus, TRAP is associated with adverse birth outcomes.

## Supplementary Material

Web_Material_kwae120

## Data Availability

Scripts for implementing the developed methods, including exposure assessment, upwind/downwind matching, and statistical analyses, are available at the GitHub repository https://github.com/larkinandy/Matching_HEI_4970. The authors are unable to share exposure assessment and epidemiologic data sets inextricably linked to residential address.
