# Supplementary material for: High traffic roads and adverse birth outcomes: comparing births upwind and downwind of the same road
Source: Am J Epidemiol. 2024 Jun 14;193(12):1720–8. doi: 10.1093/aje/kwae120 (PMC11637513; doi:10.1093/aje/kwae120)
Supplement: Web_Material_kwae120 [file web_material_kwae120.docx]

**Supplemental Material**

**High Traffic Roads and Adverse Birth Outcomes: Comparing Births Upwind and Downwind of the Same Road**

Andrew Larkin, Mary D. Willis, Lena Harris, Beate Ritz, Elaine L. Hill, Perry Hystad

**Table of Contents:**

[Figure S1. Relationship between cohort and matched pair sample sizes. 1](#_Toc165620745)

[Appendix S1: Generating annual Texas building footprints. 2](#_Toc165620746)

[Equations S1-S6: Creating tree and building shielding estimates. 3](#_Toc165620747)

[Table S1: Descriptive statistics for matched pairs, restricted to 37 to 42 weeks. 5](#_Toc165620748)

[Table S2: Relationships between wind direction and adverse birth outcomes stratified by distance to nearest road. 6](#_Toc165620749)

[Table S3. Associations between birth outcomes and living downwind of high-traffic roads, stratified by select socio-demographics. 7](#_Toc165620750)

[Table S4. Comparison of sample size and match criteria thresholds on the associations between birth outcomes and living downwind of high-traffic roads. 8](#_Toc165620751)

[Table S5. Continuous wind variables and associations between birth outcomes and living downwind of high-traffic roads 9](#_Toc165620752)

[Table S6. Associations between birth outcomes and living downwind of high-traffic roads with shielding included as a covariate. 10](#_Toc165620753)

[Table S7. Relationships between living downwind of high-traffic roads and birth outcomes, stratified by three-year running averages. 11](#_Toc165620754)

[Figure S2: Relationship between term birth weight and distance to nearest road in the upwind and downwind cohorts. 12](#_Toc165620755)

[Figure S3. Relationship between distance to a road and the likelihood of being downwind of a road. 13](#_Toc165620756)

**
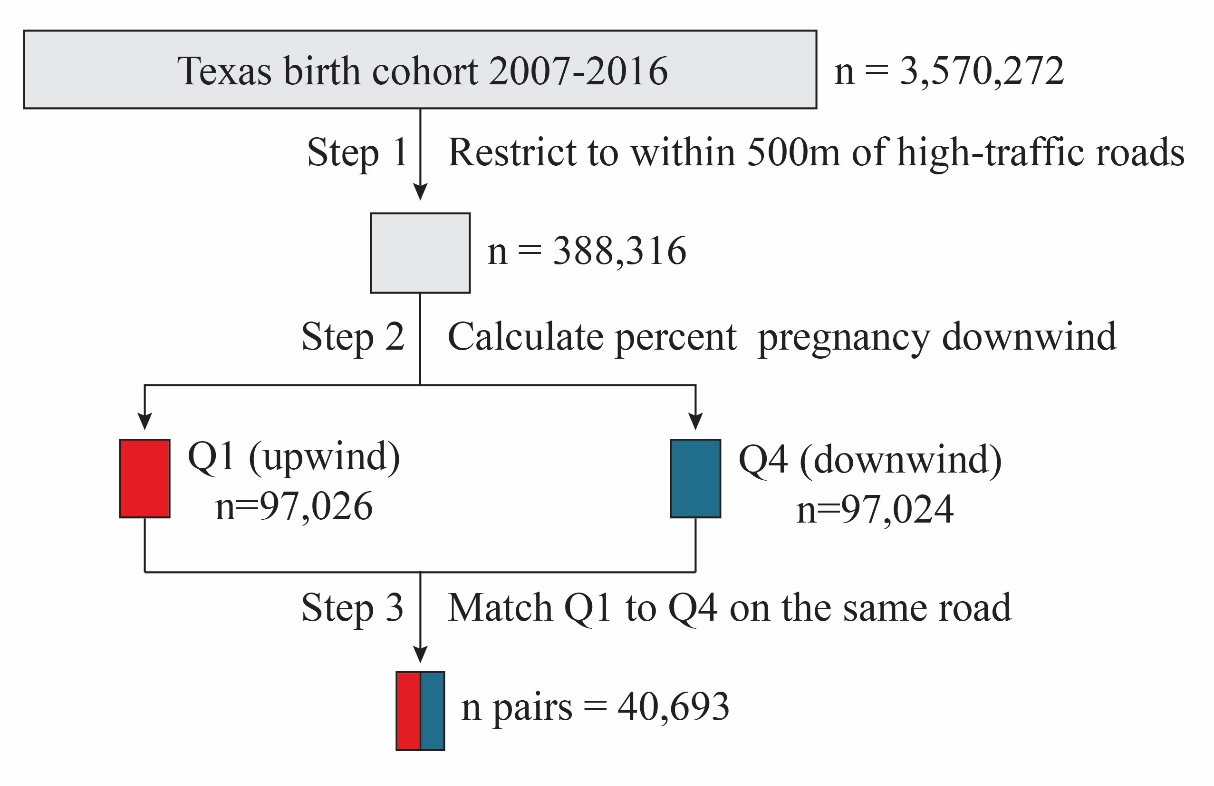
**

# **Figure S1**. Relationship between cohort and matched pair sample sizes.

# Appendix S1: Generating annual Texas building footprints.

Summary: This section provides additional details of our methodology for combining Microsoft Bing building footprints), land parcel records from the Texas Natural Resource Information System (<https://tnris.org/stratmap/land-parcels/>) and Core Logic to create annual Texas building footprints from 2007-2016. The purpose of joining these datasets was to estimate the year each building in a building footprint was built, allowing us to retrospectively create annual building footprints from 2007-2016 by removing buildings built after the year of interest.

*Dataset preprocessing.* Microsoft Bing Texas building footprints, derived from 2019 arial imagery, were downloaded from the Github repo <https://github.com/microsoft/USBuildingFootprints> in .geojson format (n footprints = 10,678,921). The building footprint file was then converted into .shp format and restricted to within 500m of maternal residences (n footprints = 5,627,589). Texas Parcel records for 2020 were downloaded by county from the Texas Natural Resources Information System. County records were then merged into a single shapefile using ArcGIS Pro (n parcels = 12,338,790) and then restricted to within 500m of maternal residences (n parcels = 5,826,557). Building characteristics are drawn from CoreLogic, a database which compiles annual property tax record information on residential housing units. The data contains a unique unit identifier, location coordinates, and characteristics including year built, type of construction, and number of stories. We pooled the annual data into an unbalanced panel dataset covering 1995 to 2020. We rolled forward any missing characteristics in a year based on the most recent previously observed value.

*Assigning Year Built to Building Footprints*. Building footprints were spatially joined with land parcels (left join). If land parcels contained only one building footprint, then the building footprint was assigned year built from the parcel records. If parcels contained more than one building footprint, buildings were spatially joined to the nearest CoreLogic point within parcel boundaries and assigned year built from the CoreLogic records.

*Creating Annual Building Footprints.* Annual building footprints for 2007-2016 were created by selecting building footprints built before the year of interest (indicated by the year built variable) in ArcGIS Pro.

# Equations S1-S6: Creating tree and building shielding estimates.

$building shielding_{li}=\frac{100 x building area_{ji}}{area_{ji}}$ Eq. S1

Where

building shielding_li_ = percent area between road segment l and maternal residence I occupied by a building footprint

building area_ji_ = area between road segment l and maternal residence I occupied by a building footprint

area_ji_ = area between road segment l and maternal residence I

$mean building shielding_{i}= \frac{1}{n}\sum_{l=i}^{n} Building Shielding_{li}$ Eq. S2

Where

n_i_ = number of high-traffic road segments within 500m of maternal residence i

l_i_ = lth 10m high-traffic road segment within 500m of maternal residence i

$Max building shielding_{i}= max(\left\{ Building Shielding_{li} \right\})$ Eq. S3

Where

{building shielding_li_} is the set of building shielding estimates for all road segments l within 500m of maternal residence i

Tree shielding estimates were derived from NASA global tree cover remote sensing estimates (30m resolution, (1)) (Eq S4-S6).

$tree shielding_{li}=\frac{1}{q}\sum_{p=1}^{q} percent tree cover_{p}$ Eq. S4

Where

tree shielding_li_ = mean percent tree cover between road segment l and maternal residence i

q = number of pixels between road segment l and maternal residence i

p = p^th^ pixel between road segment l and maternal residence i

percent tree cover_p_ = percent tree cover for pixel p

$mean tree shielding_{i}= \frac{1}{n}\sum_{l=i}^{n} tree shielding_{li}$ Eq. S5

Where

n_i_ = number of high-traffic road segments within 500m of maternal residence i

l_i_ = lth 10m high-traffic road segment within 500m of maternal residence i

$max tree shielding_{i}= max(\left\{ tree shielding_{li} \right\})$ Eq. S6

Where

{tree shielding_li_} is the set of tree shielding estimates for all road segments l within 500m of maternal residence i

# Table S1: Descriptive statistics for matched pairs, restricted to 37 to 42 weeks.

| Paired Matches - Restricted 37 to 42 Weeks (n=37631) | | |
| --- | --- | --- |
|  | paired exposed | paired control |
| downwind from max road(%) | 24.54 | 6.99 |
| mean downwind (%) | 11.29 | 4.58 |
| tree shielding (%) | 4.08 | 4.06 |
| building shielding (%) | 10.79 | 10.28 |
| estimated gestational age (wk) | 38.9 | 38.88 |
| birth weight (g) | 3329.58 | 3341.98 |
| low term birth weight (n) | 1023 | 1026 |
| preterm birth (n) | 0 | 0 |
| very preterm birth (n) | 0 | 0 |
| White (%) | 64.47 | 66.66 |
| Black (%) | 14.97 | 13.62 |
| Native American (%) | 0.23 | 0.16 |
| Asian (%) | 4.77 | 5.37 |
| Pacific Islander (%) | 0.13 | 0.17 |
| Other (%) | 14.39 | 13.09 |
| Non-Hispanic/Latina (%) | 42.77 | 44.21 |
| Hispanic (%) | 57.23 | 55.79 |
| less than 8th Grade (%) | 6.99 | 6.37 |
| Up to High School Diploma (%) | 51.21 | 48.21 |
| Up to Bachelor's Degree (%) | 35.67 | 37.97 |
| More than a Bachelor's Degree (%) | 6.05 | 7.34 |
| no reported smoking | 94.93 | 95.1 |
| reported smoking | 5.07 | 4.9 |
| low neighborhood income tertile | 33.36 | 33.33 |
| middle neighborhood income tertile | 33.31 | 33.34 |
| high neighborhood income tertile | 33.33 | 33.32 |

# Table S2: Relationships between wind direction and adverse birth outcomes stratified by distance to nearest road.

| **Term Low Birth Weight** | | | |
| --- | --- | --- | --- |
| **distance (m)** | **pairs (n)** | **events (n)** | **full model** |
| 0 to 50 | 1846 | na | -36.33 (-67.74, -4.93) |
| 51 to 100 | 4377 | na | -17.59 (-36.32, 1.15) |
| 101 to 300 | 23205 | na | -13.97 (-22.15, -5.79) |
| 301 to 400 | 14307 | na | -15.55 (-24.97, -4.13) |
| 401 to 500 | 7160 | na | 4.02 (-10.66, 18.71) |
| **Low Term Birth Weight** | | | |
| **distance (m)** | **pairs (n)** | **events (n)** | **full model** |
| 0 to 50 | 1846 | 88 | 1.95 (0.58, 6.51) |
| 51 to 100 | 4377 | 197 | 1.44 (0.91, 2.29) |
| 101 to 300 | 23205 | 1305 | 1.03 (0.89, 1.18) |
| 301 to 400 | 14307 | 770 | 0.93 (0.77, 1.12) |
| 401 to 500 | 7160 | 381 | 0.96 (0.72, 1.26) |
| **Preterm Birth** | | | |
| **distance (m)** | **pairs (n)** | **events (n)** | **full model** |
| 0 to 50 | 2048 | 391 | 1.25 (0.92, 1.69) |
| 51 to 100 | 4911 | 904 | 1.18 (0.99, 1.40) |
| 101 to 300 | 25919 | 4763 | 1.05 (0.98, 1.13) |
| 301 to 400 | 15959 | 2952 | 1.04 (0.95, 1.14) |
| 401 to 500 | 7894 | 1392 | 0.99 (0.87, 1.14) |
| **Very Preterm Birth** | | | |
| **distance (m)** | **pairs (n)** | **events (n)** | **full model** |
| 0 to 50 |  |  | did not converge |
| 51 to 100 | 4911 | 126 | 3.68 (1.71, 7.90) |
| 101 to 300 | 25919 | 673 | 1.14 (0.91, 1.43) |
| 301 to 400 | 15959 | 461 | 1.11 (0.81, 1.52) |
| 401 to 500 | 7894 | 223 | 1.01 (0.61, 1.67) |

# Table S3. Associations between birth outcomes and living downwind of high-traffic roads, stratified by select socio-demographics.

|  | **Birth Weight** | | **Low Term Birth Weight** | | **Premature Birth** | | | **Very Premature Birth** | |
| --- | --- | --- | --- | --- | --- | --- | --- | --- | --- |
|  | **pairs (n)*** | **Difference (g)** | **events (n)** | **Odds Ratio** | **pairs (n)**** | **events (n)** | **Odds Ratio** | **events (n)** | **Odds Ratio** |
| **Black non-Hispanic** | 1634 | 20.70 (-10.80, 52.21) | 164 | 1.21 (0.69, 2.12) | 1935 | 474 | 1.21 (0.94, 1.56) | 111 | 2.92 (1.00, 8.53) |
| **White non-Hispanic** | 3722 | -5.93 (-25.77, 13.87) | 131 | 0.87 (0.48, 1.58) | 4019 | 559 | 1.05 (0.77, 1.18) | 72 | did not converge |
| **Hispanic or Latina** | 10020 | -8.02 (-20.41, 4.37) | 597 | 0.99 (0.80, 1.24) | 11203 | 2016 | 1.02 (0.88, 1.10) | 344 | 1.18 (0.82, 1.73) |
| **High school** | 3731 | -23.02 (-43.51, -2.54) | 220 | 1.85 (1.18, 2.88) | 4184 | 810 | 1.11 (0.92, 1.34) | 124 | 2.69 (0.52, 13.88) |
| **Gt than high school** | 20488 | -8.70 (-17.32, -0.08) | 1062 | 0.97 (0.83, 1.12) | 22758 | 3995 | 0.99 (0.92, 1.07) | 598 | 1.02 (0.79, 1.32) |
| **US born** | 17105 | -12.87 (-22.23, -3.50) | 1062 | 0.98 (0.84, 1.14) | 19311 | 3750 | 0.99 (0.91, 1.07) | 576 | 1.06 (0.82, 1.36) |
| **Foreign born** | 5475 | -8.63 (-25.89, 8.63) | 232 | 0.92 (0.60, 1.42) | 5926 | 846 | 1.16 (0.96, 1.39) | 114 | 1.23 (0.39, 3.87) |
| **Low income** | 7405 | 1.73 (-12.42, 15.88) | 455 | 1.04 (0.79, 1.35) | 8218 | 1587 | 1.14 (1.01, 1.28) | 258 | 1.15 (0.75, 1.74) |
| **High income** | 7742 | -13.59 (-27.55, 0.36) | 368 | 0.77 (0.58, 1.03) | 8609 | 1406 | 0.97 (0.85, 1.11) | 207 | 0.53 (0.29, 0.95) |

*number of pairs are the same for birth weight and low term birth weight. **number of pairs are the same for premature birth and very premature birth

# Table S4. Comparison of sample size and match criteria thresholds on the associations between birth outcomes and living downwind of high-traffic roads.

| **Term Low Birth Weight** | | | | |
| --- | --- | --- | --- | --- |
| **match criteria** | **pairs (n)** | **events (n)** | **base model** | **full model** |
| 15 | 10293 | na | -18.04 (-29.87, -6.21) | -15.70 (-28.02, -3.39) |
| 25 | 16117 | na | -18.00 (-27.45, -8.54) | -12.68 (-22.45, -2.92) |
| 50 | 25997 | na | -15.31 (-22.75, -7.86) | -12.12 (-19.81, -4.42) |
| 100 | 37631 | na | -13.83 (-20.03, -7.63) | -11.61 (-18.01, -5.21) |
| **Low Term Birth Weight** | | | | |
| **match criteria** | **pairs (n)** | **events (n)** | **base model** | **full model** |
| 15 | 10293 | 584 | 1.09 (0.91, 1.31) | 1.08 (0.87, 1.34) |
| 25 | 16117 | 899 | 1.18 (1.02, 1.36) | 1.21 (1.02, 1.44) |
| 50 | 25997 | 1449 | 1.07 (0.96, 1.20) | 1.04 (0.91, 1.18) |
| 100 | 37631 | 2081 | 1.00 (0.91, 1.10) | 0.98 (0.88, 1.09) |
| **Preterm Birth** | | | | |
| **match criteria** | **pairs (n)** | **events (n)** | **base model** | **full model** |
| 15 | 11809 | 2204 | 0.97 (0.88, 1.07) | 0.98 (0.88, 1.08) |
| 25 | 18332 | 3368 | 1.06 (0.99, 1.14) | 1.05 (0.97, 1.14) |
| 50 | 29192 | 5320 | 1.04 (0.98, 1.11) | 1.04 (0.97, 1.11) |
| 100 | 41912 | 7684 | 1.01 (0.96, 1.06) | 1.01 (0.96, 1.07) |
| **Very Preterm Birth** | | | | |
| **match criteria** | **pairs (n)** | **events (n)** | **base model** | **full model** |
| 15 | 11809 | 348 | 1.01 (0.79, 1.30) | 1.08 (0.73, 1.60) |
| 25 | 18332 | 509 | 1.03 (0.84, 1.26) | 1.17 (0.86, 1.57) |
| 50 | 29192 | 824 | 0.98 (0.84, 1.14) | 1.07 (0.86, 1.33) |
| 100 | 41912 | 1153 | 0.96 (0.84, 1.09) | 1.03 (0.87, 1.23) |

# Table S5. Continuous wind variables and associations between birth outcomes and living downwind of high-traffic roads

| **outcome** | **pairs (n)** | **events (n)** | **full model** | **percent pregnancy downwind (10%)** |
| --- | --- | --- | --- | --- |
| term birth weight (g) | 37631 | NA | -11.61 (-18.01, -5.21) | -4.96 (-8.43, -1.50) |
| low term birth weight (OR) | 37631 | 2049 | 0.98 (0.88, 1.09) | 0.99 (0.99, 1.00) |
| preterm birth (OR) | 40693 | 7511 | 1.01 (0.96, 1.07) | 1.00 (1.00, 1.01) |
| very preterm birth (OR) | 40693 | 1114 | 1.03 (0.87, 1.23) | 1.01 (1.00, 1.01) |

# Table S6. Associations between birth outcomes and living downwind of high-traffic roads with shielding included as a covariate.

| **Outcome** | **pairs (n)** | **events (n)** | **full model** | **shielding model** |
| --- | --- | --- | --- | --- |
| term birth weight (g) | 37631 | NA | -11.61 (-18.01, -5.21) | -11.61 (-18.02, -5.20) |
| low term birth weight (OR) | 37631 | 2049 | 0.98 (0.88, 1.09) | 0.97 (0.87, 1.08) |
| preterm birth (OR) | 40693 | 7511 | 1.01 (0.96, 1.07) | 0.99 (0.96, 1.07) |
| very preterm birth (OR) | 40693 | 1114 | 1.03 (0.87, 1.23) | 1.04 (0.88, 1.24) |

Shielding model: full model + max tree and max building shielding

# Table S7. Relationships between living downwind of high-traffic roads and birth outcomes, stratified by three-year running averages.

| **Term Birth Weight** | | | | |
| --- | --- | --- | --- | --- |
| **3 year models** | **pairs (n)** | | **Events (n)** | **full model** |
| 2007-2009 | 15928 | | na | -11.44 (-21.37, -1.50) |
| 2008-2010 | 18264 | | na | -7.55 (-16.87, 1.78) |
| 2009-2011 | 19979 | | na | -4.81 (-13.76, 4.14) |
| 2010-2012 | 20350 | | na | -9.52 (-18.43, -0.60) |
| 2011-2013 | 21538 | | na | -19.15 (-27.79, -10.51) |
| 2012-2014 | 20239 | | na | -14.69 (-23.57, -5.82) |
| 2013-2015 | 16829 | | na | -15.52 (-25.22, -5.82) |
| 2014-2016 | 13070 | | na | -21.15 (-32.07, -10.23) |
| **Low Term Birth Weight** | | | | |
| **3 year models** | | **pairs (n)** | **events (n)** | **full model** |
| 2007-2009 | | 15928 | 795 | 1.07 (0.89, 1.28) |
| 2008-2010 | | 18264 | 949 | 0.95 (0.80, 1.12) |
| 2009-2011 | | 19979 | 1037 | 0.92 (0.79, 1.08) |
| 2010-2012 | | 20350 | 1137 | 0.90 (0.77, 1.05) |
| 2011-2013 | | 21538 | 1201 | 0.96 (0.93, 1.11) |
| 2012-2014 | | 20239 | 1167 | 0.94 (0.81, 1.09) |
| 2013-2015 | | 16829 | 961 | 0.96 (0.84, 1.17) |
| 2014-2016 | | 13070 | 762 | 1.04 (0.86, 1.24) |
| **Premature Birth** | | | | |
| **3 year models** | | **pairs (n)** | **events (n)** | **full model** |
| 2007-2009 | | 17719 | 3295 | 1.06 (0.98, 1.16) |
| 2008-2010 | | 20302 | 3693 | 1.09 (1.00, 1.18) |
| 2009-2011 | | 22231 | 4106 | 1.05 (0.97, 1.13) |
| 2010-2012 | | 22439 | 4039 | 1.05 (0.97, 1.14) |
| 2011-2013 | | 23711 | 4224 | 1.00 (0.93, 1.08) |
| 2012-2014 | | 22286 | 4009 | 0.98 (0.91, 1.06) |
| 2013-2015 | | 18456 | 3352 | 1.00 (0.92, 1.09) |
| 2014-2016 | | 14451 | 2647 | 0.99 (0.90, 1.09) |
| **Very Premature Birth** | | | | |
| **3 year models** | | **pairs (n)** | **events (n)** | **full model** |
| 2007-2009 | | 17719 | 473 | 1.22 (0.92, 1.61) |
| 2008-2010 | | 20302 | 544 | 1.17 (0.90, 1.52) |
| 2009-2011 | | 22231 | 622 | 0.96 (0.76, 1.22) |
| 2010-2012 | | 22439 | 593 | 0.92 (0.72, 1.18) |
| 2011-2013 | | 23711 | 616 | 0.91 (0.72, 1.17) |
| 2012-2014 | | 22286 | 614 | 0.95 (0.74, 1.21) |
| 2013-2015 | | 18456 | 529 | 1.10 (0.85, 1.43) |
| 2014-2016 | | 14451 | 418 | 1.21 (0.90, 1.63) |


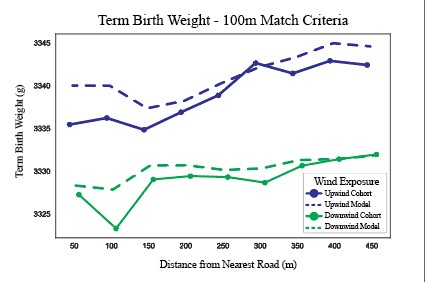


# Figure S2: Relationship between term birth weight and distance to nearest road in the upwind and downwind cohorts.


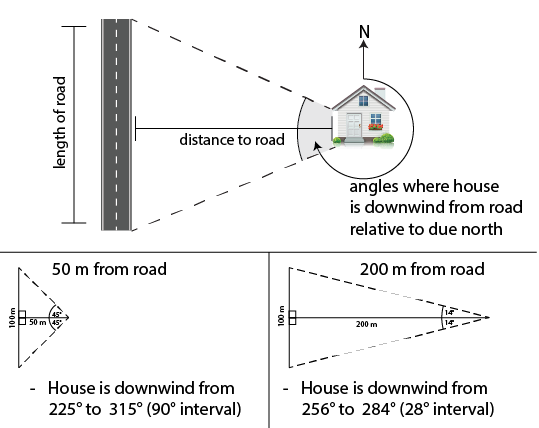


Figure S3. Relationship between distance to a road and the likelihood of being downwind of a road. Residences closer to roads have a greater range of radial intervals where there are downwind from a road segment.

**References**

1. Sexton JO, Song XP, Feng M, Noojipady P, Aand A, Huang C, et al. Global, 30-m resolution continuous fields of tree cover: Landsat-based rescaling of MODIS vegetation continuous fields with lidar-based estimates of error. Intern J Health. 2013 Sep 1;6(5):427-448.
